# ERP Evidences of Rapid Semantic Learning in Foreign Language Word Comprehension

**DOI:** 10.3389/fnins.2022.763324

**Published:** 2022-03-03

**Authors:** Akshara Soman, Prathibha Ramachandran, Sriram Ganapathy

**Affiliations:** Learning and Extraction of Acoustic Patterns Lab, Indian Institute of Science, Bangalore, India

**Keywords:** ERPs, language learning, semantics, word learning, speech perception

## Abstract

The event-related potential (ERP) of electroencephalography (EEG) signals has been well studied in the case of native language speech comprehension using semantically matched and mis-matched end-words. The presence of semantic incongruity in the audio stimulus elicits a N400 component in the ERP waveform. However, it is unclear whether the semantic dissimilarity effects in ERP also appear for foreign language words that were learned in a rapid language learning task. In this study, we introduced the semantics of Japanese words to subjects who had no prior exposure to Japanese language. Following this language learning task, we performed ERP analysis using English sentences of semantically matched and mis-matched nature where the end-words were replaced with their Japanese counterparts. The ERP analysis revealed that, even with a short learning cycle, the semantically matched and mis-matched end-words elicited different EEG patterns (similar to the native language case). However, the patterns seen for the newly learnt word stimuli showed the presence of P600 component (delayed and opposite in polarity to those seen in the known language). A topographical analysis revealed that P600 responses were pre-dominantly observed in the parietal region and in the left hemisphere. The absence of N400 component in this rapid learning task can be considered as evidence for its association with long-term memory processing. Further, the ERP waveform for the Japanese end-words, prior to semantic learning, showed a P3a component owing to the subject's reaction to a novel stimulus. These differences were more pronounced in the centro-parietal scalp electrodes.

## 1. Introduction

Humans have the unique ability to learn and process languages throughout ones life time. The main hypothesis for learning a new language states that the process begins by imitation of the sounds spoken by native speakers of the language (Speidel and Nelson, 2012). The association of semantics with speech sounds constitutes the next phase of learning (Clark, 1973), which further develops to sentence formation and syntax/grammar learning. These processes may not be sequential and may be interleaved with each other.

The brain activity related to the perception and cognition of language can be studied through event-related potentials (ERPs). The ERPs are computed by averaging the electroencephalogram (EEG) recordings evoked by the same event. The ERPs triggered by verbal stimuli have been associated with different aspects of language learning (Kutas and Hillyard, 1984).

In this article, we present a study to analyze rapid language learning effects using ERP analysis where the end-words of English sentences were replaced with Japanese words. In the first phase of the experiment, the subjects who were proficient in English with no prior exposure to Japanese words were presented with English sentences containing Japanese end-words. A subsequent language learning phase introduces the semantics of the Japanese stimuli through its pictorial description. In the final phase, the subjects listen to English sentences again with Japanese end-words armed with the knowledge of the semantics. The Japanese end-words in the English sentences may be congruent or in-congruent with the sentence context. An ERP analysis on Japanese end-words before and after semantic learning illustrate significant changes that highlight the neural processes involved in learning. Further, the difference ERP of Japanese stimuli in congruent and incongruent condition (after language learning) elicits delayed and positive ERP components as opposed to the N400 effects observed in native language under semantic mis-match condition.

**Contributions:**

- The study contrasts the ERP effects of semantic incongruity in a proficient language vs. a newly acquired language.
- This study demonstrates that rapid semantic learning elicited ERP responses in the form of a delayed positive response at 500–700 ms from the onset of the end-word for the incongruent words.
- The scalp electrodes showing semantic effects from a newly learned word of a foreign language are located in the pareital and occipital regions.
- The amplitude of semantic incongruity (ERP component) in foreign language (Japanese) is more for pure foreign words (hiragana words in Japanese) vs. English loan words (katakana words in Japanese).

## Relevant Literature

The N400 component was first introduced by Kutas and Hillyard (1980), where the reading task composed of presenting the participant with a set of sentences that end with a congruent or incongruent word. These semantically incongruent end-words in a sentence elicited specific type of ERPs (Nobre and Mccarthy, 1995; Hoeks et al., 2004; Dikker and Pylkkanen, 2011), known as the N400, a negative-going deflection between 250 and 400 ms after the end-word onset. The presence of N400 in semantically incongruent stimuli was also observed in other stimuli presentations (Kutas et al., 1987) like reading (Szűcs et al., 2007) and visual forms (Nigam et al., 1992). The N400 was characterized as a reaction to an unexpected or inappropriate, but syntactically correct word at the end of a sentence. The N400 component was not observed for stimuli with syntactical and grammatical errors (Kutas and Hillyard, 1983). This result is seen as the evidence that the N400 wave is more closely related to the semantics than to the syntactical processing.

The N400 is not only a response to semantic improbability or anomaly, but also as an indicator of the access to semantic information associated with the stimuli (Kutas and Federmeier, 2011). When a word is congruent in its context, there is little new information to process and hence, this evokes lower N400 response than an incongruent word. The amplitude of the N400 is sensitive to a word's semantic expectancy (Brown and Hagoort, 1993) and found to be larger in response to more unexpected stimuli (Curran et al., 1993; Holcomb, 1993; DeLong et al., 2005). The N400 has also been used to show that language comprehension is incremental (Van Petten and Kutas, 1990) and involves prediction (Federmeier and Kutas, 1999b). Further, semantic information processing happens even without active awareness (Luck et al., 1996). It has also been pointed out that language mechanisms vary across the hemispheres (Federmeier and Kutas, 1999a) and can change over the course of normal aging (Wlotko et al., 2010).

Even though the N400 has contributed significantly to the understanding of language comprehension, the N400 response is not confined to language domain alone, and hence it is not a “language-component” (Leckey and Federmeier, 2020). The N400 is not only seen in word comprehension, but also for different kinds of pictorial stimuli (e.g., comics/cartoons, drawings, pictures of objects, natural scenes), faces, gestures, and environmental sounds. Thus, it can be elicited for any kind of stimulus linked to long-term memory representations (Kutas and Federmeier, 2011).

The P3a, or novelty P3 (Comerchero and Polich, 1999), is a positive-going component with peak latency in the range of 250–280 ms. It is topographic distribution that shows maximum amplitude over frontal and central electrode sites. P3a has been associated with cognitive tasks of involuntary attention and the processing of novelty (Polich, 2007).

### 1.1. N400 as an Index of Word Learning

The N400 has been established in numerous studies to be a useful index of new word learning. In a study by Perfetti et al. (2005), adults were taught the meanings of infrequent and unfamiliar words. The N400 component was seen for unrelated word pairings containing the trained words and not for those involving the unfamiliar words.

Mestres-Missé et al. (2007) investigated context-based learning of novel words using ERPs. The researchers specifically introduced novel word forms in the ending position of meaningful sentences that the participants read during the training phase. In a following relatedness judgment task on word pairs, consisting of a trained novel word (prime) and a real word (target), the study found a reduction in the N400 for targets words that were associated with the prime word compared to the unrelated target-prime pairs.

Batterink and Neville (2011) investigated contextual learning by embedding pseudo-words into meaningful short story contexts. In a subsequent relatedness judgment task, the study showed a reduction in the N400 amplitude for targets corresponding to the novel word.

The N400 has also been demonstrated to be sensitive to new word learning after just one exposure to a novel word in the context of a highly predictive sentence (Borovsky et al., 2010, 2012). The findings of such investigations provide neuro-physiological evidence for understanding the semantic learning of the new words.

### 1.2. Intra-Sentential Code-Switching

The N400, left-lateralized anterior negativity (LAN), and the late positive component (LPC; also referred to as P600) are the three primary ERP components identified in research on intra-sentential code switching.

The LAN is a left-lateralized anterior negativity that occurs in the same time frame as the N400 (300–500 ms) but with a distinct topographic scalp distribution. Friederici (2002) observed LAN effects in morphosyntactic processing, as well as in the processing of code-switched sentences. The higher working memory load resulting from integrating morphological signals of the code-switched word into the wider sentence context was interpreted as the switch-related LAN component (Moreno et al., 2002).

The LPC (or P600) is a positive-going wave that arises 500–600 ms after the stimulus and lasts several hundred milliseconds (Osterhout and Holcomb, 1992; Hagoort et al., 1993). It has a wide posterior scalp distribution and is strongest in the centro-parietal areas. Friederici (1995), Kaan et al. (2000), and Tanner et al. (2017) infer that the LPC indexes sentence-level rearrangement or re-analysis. The LPC, according to this view, indicates sentence-level wrap-up or meaning revision process, which in the instance of intra-sentential code-switching, reflects the sentence-level reorganization of two languages into a cohesive utterance. The LPC has also been linked to the processing of unexpected or unlikely task-relevant events (McCallum et al., 1984; Coulson et al., 1998), as well as the reorganization of stimulus-response mapping (Moreno et al., 2008). A switch-related LPC represents bilinguals' perception of a language transition as an unexpected occurrence involving a shift in form rather than meaning (Moreno et al., 2002).

## 2. Materials and Methods

**Figure 1:**
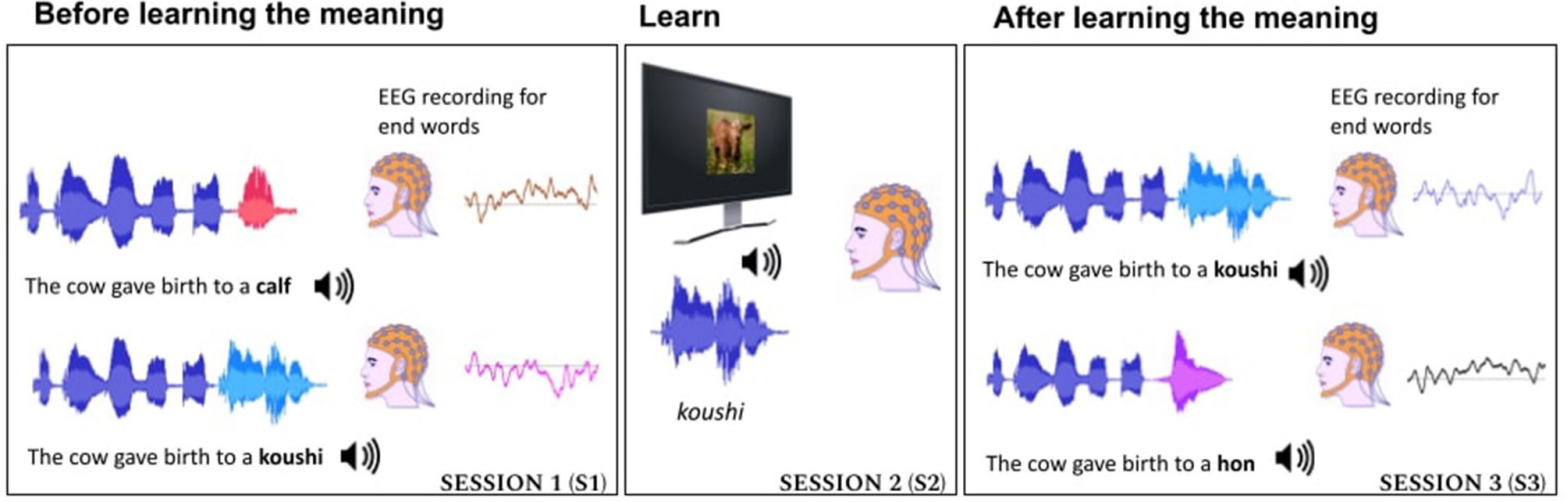
Experiment pipeline consisted of three sessions. S1—where the subject listened to acoustics of Japanese words used in English context and English end-words in congruent and incongruent context. S2—where the semantics of Japanese words were introduced using images. S3—where the subject listened to Japanese words in English context (both congruent sentences and incongruent sentences).

The experimental paradigm used in this study is illustrated in Figure 1.

### 2.1. Stimuli

All the speech stimuli used in the study were recorded from speech provided by a single female speaker who was proficient in both English and Japanese languages. The speaker's first language was Tamil, a south-Indian language. The accent of the speaker was Indian English. Given that all the listeners were also from Indian origin, we found that the listeners had no issues in understanding the English content. Further, the work had employed a single speaker for all the stimuli. This helped to remove the effect of speaker variabilities or speaker switching in the stimuli used.

The speech stimuli used in the experiment consisted of isolated sentences and isolated words. They were recorded in a sound proof booth. The entire stimuli set used in the experiment is listed in Supplementary Table 2. The audio and image files used for the experiment are available in this project's GitHub repository^1^. The stimuli set consisted of 90 unique English sentences. The sentences were selected such that the end-word was highly predictable from the sentence context. Our stimuli set was taken from the high cloze probable sentences (cloze probability in the range of 67–100%) of the Block-Baldwin sentence set (Block and Baldwin, 2010). The audio duration of the sentence varied between 1.4 and 2.4 s with an average of 2.2 s.

The end-words of the sentences were either from English or Japanese language and they were designed to be either semantically congruent or incongruent to the preceding context in the sentence. All the stimuli conditions are listed in Table 1. The first condition (C1) consists of 90 English sentences from the original Block-Baldwin set without any modification. The stimuli for the other three conditions of the experiment were created by replacing the end-word with all the preceding words intact. In condition 2 (C2), the end-word was replaced by an English word which is unexpected in the sentence context (English incongruent condition); in condition 3 (C3), the end-word was replaced by a Japanese word of the congruent semantics (Japanese—congruent condition); and in condition 4 (C4), the end-word was replaced by a Japanese word of unexpected meaning (Japanese—incongruent condition). Thus, the experiment had a total of 360 sentences. The sentences were carefully chosen such that the end-word can be visualized as an image. Each of the stimuli condition used the same base set of English sentences. The conditions differed only in terms of the end-word of the sentences. Each stimulus was recorded as a full sentence for each condition separately.

**Table 1:** Different conditions of the stimuli sentences used in our experiment with an example.

**Stimuli condition**	**Example**
C1—Eng. Congruent	*The cow gave birth to a **calf***.
C2—Eng. Incongruent	*The cow gave birth to a **book***.
C3—Jap. Congruent	*The cow gave birth to a **koushi***.
C4—Jap. Incongruent	*The cow gave birth to a **hon***.

*Endword of the stimulus sentence is shown in bold. Different endwords give rise to different stimuli conditions*.

We choose the Japanese language as the novel language as it does not belong to the same language family as English. At the same time, Japanese language contains a set of loan-words from English termed as katakana words. The katakana words are typically English words that have been adapted without translation into the Japanese language (Olah, 2007). The katakana words sound similar to their English counterparts (cognate words). By using a mix of non-cognate native Japanese words (referred to as hiragana words) and katakana words, we were able to study the effect of phonetic similarity in learning. The set of Japanese words used in our experiments (Supplementary Table 2) consisted of 38 katakana words and 52 hiragana Japanese words. This unbalanced distribution of katakana and hiragana words are in compliance with the word frequency distribution in the Japanese language (Chikamatsu et al., 2000). Thus, for stimuli conditions C3 and C4, the end-word can be either a katakana word or a hiragana word.

### 2.2. Experiment Flow

A schematic illustration of the experiment is shown in Figure 1. A short video depicting the experiment flow is available in this project's GitHub repository^2^.The end-words were either congruent or incongruent to the context of the sentence. The participants learned the semantics of the unknown Japanese words using image supervision (shown in session 2 of Figure 1) and the learning was analyzed when the words were used in sentences both in congruent and incongruent condition (shown in session S3 of Figure 1). In each session, the sentences of different conditions were presented in random order using a loud speaker.

In session S1, the subject listened to English sentences with congruent or incongruent end-word (C1 and C2 from Table 1). This session also contained English sentences with Japanese end-word. The subject got the first exposure to these Japanese words in this session without the semantic information.

In the next session (S2), the participants were provided with the semantics of the Japanese words. A block of five new Japanese words was considered and word meanings were conveyed using the respective image. The image form of the word and its audio are presented simultaneously. The five words of each block were presented in random order. A retrieval task was also designed to ensure the learning ability of the subject. In the retrieval task, the subject was asked to speak the Japanese word for the image shown in the computer screen. After the subject provided the spoken response, the audio of the correct Japanese word was replayed. Here, the subject was affirming the learning or corrected their learning if the word recollection was inaccurate. We do not analyze the EEG data from S2 in this article.

In session 3 (S3), the subject listened to the sentence audio played through the loudspeaker. It was an English sentence with a Japanese end-word. Here, the end-word was either congruent or incongruent to the context of the sentence (C3 and C4 in Table 1). The end-word was one of the 5 Japanese words learned in the preceding session (S2). Hence, there were a total of 10 sentences (equal number of congruent and incongruent) played in session 3 for the current block. These 10 sentences were presented in random order. After the audio signal was played, a recognition task was carried out to ensure that the subject recollected the meaning of the Japanese end-word. In the recognition task, the subject was asked to pick the image corresponding to the Japanese end-word. The subjects recorded their choice of image by speaking the corresponding number index on the screen. The subject responses were later evaluated manually to assess their recognition accuracy. The behavioral results are discussed in Section 3.1. Only the EEG responses to the Japanese words, whose meaning was recollected correctly, were used in the subsequent ERP analysis.

In order to avoid the memory load of learning and recalling 90 new Japanese words, S2 and S3 were performed in 18 blocks of five words each. The session S3 for a set of five words was conducted immediately after the subject was trained on the semantics in session S2. We designed the experiment in this way to reduce the memory load on subject as we were more interested to analyze the semantic effects of the newly learned words in both congruent and incongruent condition. A particular Japanese word was presented five times to the subject: once in S1 (sentence end-word without semantic knowledge), twice in S2 (as isolated words in the learning phase) and twice in S3. In S3, it was used as the sentence end-word in congruent and incongruent context. A particular sentence and end-word pair was introduced only once in the whole experiment. For incongruent condition, sentence-end-word pairing was carried by random shuffling and incongruence was ensured by manual selection. The order of congruent and incongruent conditions in S3 was randomized. Thus, the exposure to new Japanese words were balanced across conditions. The three sessions were recorded in an interleaved fashion in one recording setup for each of the subjects. The experiment design ensured that the subject does not get exposed to katakana words more than hiragana words.

### 2.3. Participants

The participants had self-reported normal hearing and no history of neurological disorders. Twenty-one subjects took part in the experiment. Two subjects were eliminated due to poor EEG data quality and another two were eliminated due to equipment failure. Seventeen adults participated in this study (mean age = 25.7, age span = 22–35, 7 female and 10 male) and they had an intermediate or higher level of English proficiency. This was verified with the Oxford Listening Level Test^3^ before the commencement of the experiment. The native language of the subjects was one of the five Indian languages (Malayalam, Tamil, Kannada, Telugu, or Hindi). All subjects provided written informed consent to take part in the experiment and received a monetary compensation. The Indian Institute of Science Human Ethics Board approved all procedures of the experiment. The methods were carried out in accordance with the relevant guidelines and regulations.

We have performed the power analysis with an assumed effect size of d = 0.5 (Zwaan et al., 2018; Brysbaert, 2019) to check the sufficiency of the number of subjects and trials used in the experiments. We performed the power analysis on our experiment using the GPower software (Faul et al., 2007). This analysis revealed that our study is a properly powered experiment (with power value more than 95%). Hence, the effects reported in the study are very likely to be robust and reproducible.

### 2.4. EEG Recording Setup

The EEG signals were recorded employing a BESS F-64 amplifier with 64 passive gel-based Ag/AgCl electrodes placed according to the extended 10–20 positioning NeuroScan 4.5 system (Soman et al., 2019). It was recorded at a sampling rate of 1,024 Hz. An isolated frontal electrode was utilized as ground and the average of two earlobe electrodes was utilized as reference. The channel impedance was kept underneath 10 kω throughout the recording. The EEG recording took place in a sound-proof, electrically isolated room.

### 2.5. Data Preprocessing

Prior to ERP offline-averaging, the line noise was removed and the continuous EEG data were band-pass filtered between 1 and 8 Hz. The noisy trials were automatically removed using EEGLAB (Delorme and Makeig, 2004).

## 3. Results

### 3.1. Behavioral Task

**Figure 2:**
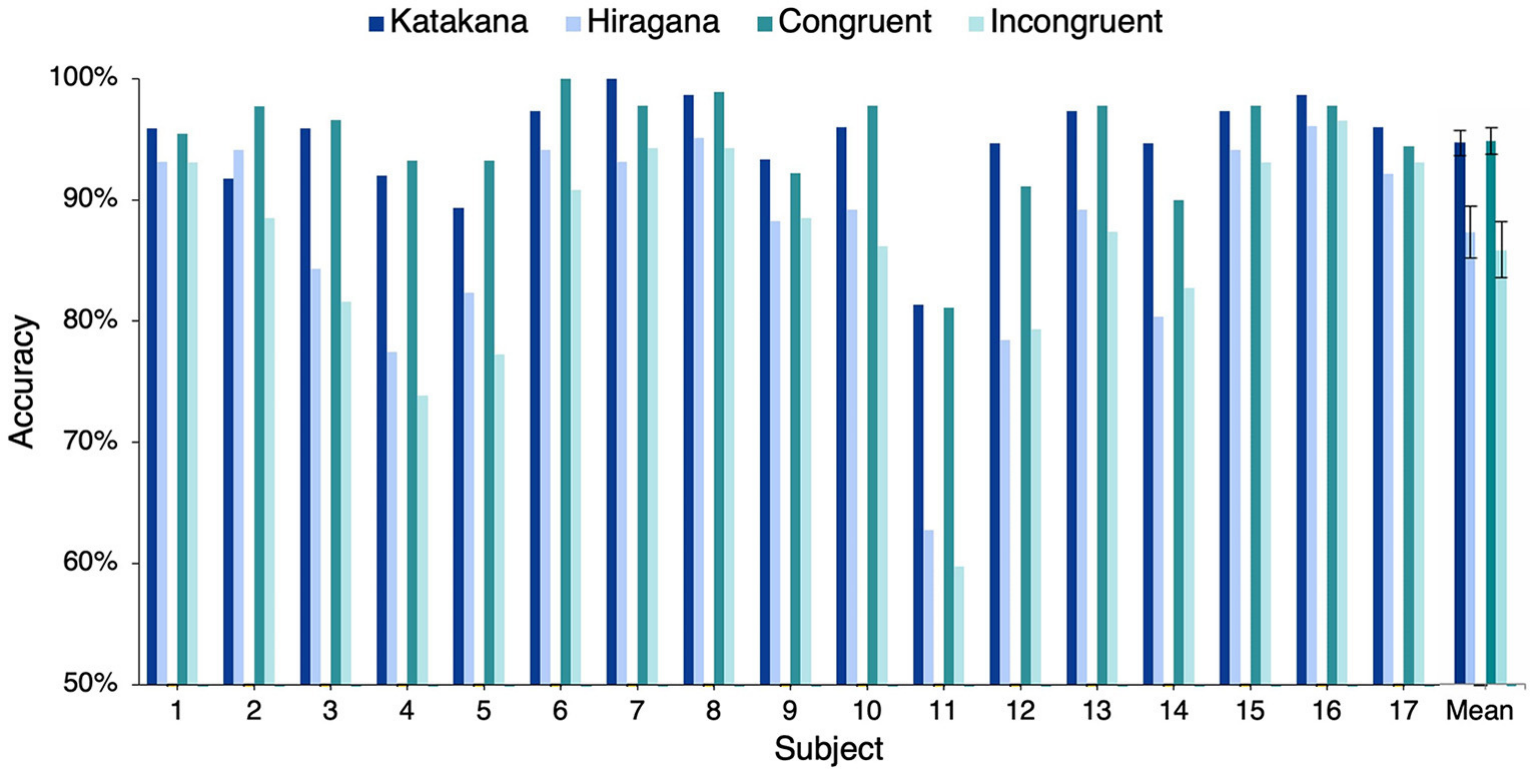
Behavioral task performance: this plot shows the percentage of Japanese words correctly associated with the image description by the subjects.

A behavioral task was conducted to ensure that the subject successfully recalled the meaning of the Japanese words while listening to the end-word of the sentence stimuli in session S3. From a set of five images, the subject was asked to identify the image corresponding to the Japanese end-word of the sentence. Figure 2 shows the percentage of words' whose meaning was correctly identified by the subjects. The solid line shows the overall accuracy obtained by each subject. Eleven of 17 subjects correctly recalled more than 90% of the word semantics (chance accuracy was 20%). Thus, the subsequent ERP-based conclusions in S3 had a strong behavioral basis. As seen in Figure 2, the number of correct responses in the Japanese congruent condition was greater than number of correct responses in the incongruent condition for all the subjects. A right-tailed paired sample *t*-test showed that recognition accuracy of congruent end-words was significantly higher than incongruent end-words [congruent: 94.88, incongruent: 85.90, *t*_(16)_ = 5.92, *p* = 1.06*e*−5]. This indicated that it was easy to recollect the meaning of a word when it was used in the correct semantic context in a sentence. Figure 2 shows that the recognition accuracy of katakana words are better than that of hiragana words for all subjects except one (subject 2). A right-tailed paired sample *t*-test showed that recognition accuracy of katakana words was significantly higher than hiragana words [katakana: 94.72, hiragana: 87.31, *t*_(16)_ = 5.32, *p* = 3.46*e*−5]. Hence, we conclude that the lexical association of katakana words with English words made it easier to recall katakana words. In the subsequent analysis, only words that were correctly recalled are used.

### 3.2. ERP Analysis

The ERPs are time-locked EEG responses averaged across multiple trials for the same stimulus condition. The ERPs are computed for epochs extending from 100 ms before the end-word onset to 800 ms after the end-word onset. The time *t* = 0 in the ERP plots corresponds to the onset of the end-word in the stimuli sentences. The difference ERP waves were calculated by subtracting an ERP wave of one condition from the other. All the grand average ERP plots shown in this paper are ERP responses averaged across 17 subjects. The two sample *t*-test was conducted at each time sample to validate the significance of the ERP responses. All difference ERP plots are marked with time regions of significance where the difference value is significantly above zero (*p* < 0.05). This is indicated by horizontal bars in the bottom of the plot.

#### 3.2.1. Effect of Incongruity

**Figure 3:**
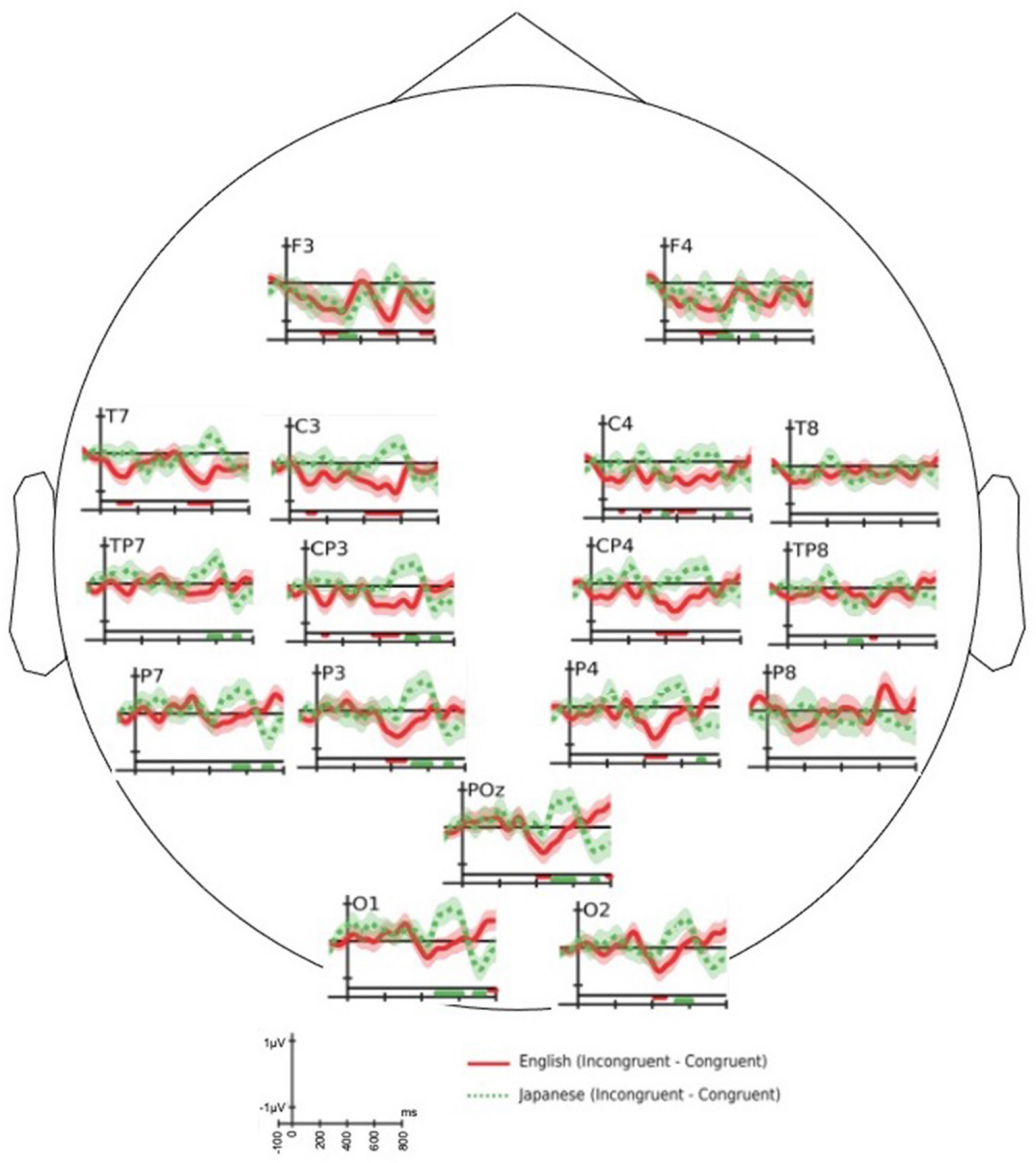
The grand average of difference event-related potential (ERP) response of congruent end-word from in-congruent end-word: end-words in English (solid—red color) and Japanese: session 3 (dotted—green color). The horizontal bars drawn in the bottom of each plot identifies the time regions with significant (two-sample *t*-test with *p* < 0.05) difference in the ERP response. The bars have the same color of the associated difference waveform.

The ERP response shown by solid line in Figure 3 exhibits N400 effect [*t*_(16)_ = −5.59, *p* = 2.05*e*−05] in 300–500 ms over centro-parietal and parietal electrodes) for the difference of English congruent response (*C1S1*) from English incongruent response (*C2S1*). This result is aligned with the prior research on N400 for auditory tasks (Hagoort and Brown, 2000), which is elicited for semantically incongruent stimuli conditions. To the best of our knowledge, the ERP analysis for other conditions that follow are reported for the first time in literature.

The difference of grand average ERP response of Japanese congruent end-word from Japanese incongruent end-word is shown in Figure 3 as dotted line. The semantic incongruity of newly learned Japanese end-words did not evoke an N400 response relative to the congruent Japanese end-words [*t*_(16)_ = 0.32, *p* = 0.75 over the time-window 300–500 ms] over the cluster of centro-parietal and parietal electrode locations.

As observed in Figure 3, the English and Japanese end-words had different responses around 400 and 600 ms. Both the English and Japanese difference ERP did not have significant peak in the early part of the time axis. The difference ERP response for Japanese end-words elicited a P600 like component [*t*_(16)_ = 5.11, *p* = 5.24*e*−05 for time-window: 500–700 ms] over a cluster of centro-parietal and parietal electrode locations. This figure also highlights that ERP effects of semantic incongruity are possible without a long-term learning process. A similar difference in ERP response for English end-words in the 500–700 ms time-window [*t*_(16)_ = −1.05, *p* = 0.310] was not observed. In other words, the semantic incongruency in the stimuli did not evoke P600 response for familiar language.

To confirm these differences statistically, we performed ANOVA on the mean ERP amplitudes across the scalp in two time-windows (300–500 and 500–700 ms) of interest. We considered congruity and scalp region (frontal/central/parietal) as the independent factors. In the N400 time-window (300–500 ms), we observed robust effects of congruity for English end-words. We observed similar effects for Japanese end-words in the P600 (500–700 ms) window. The ANOVA reveals significant interaction between language and congruity in both the time-windows [for 300–500 ms window: *F*_(1, 68)_ = 16.13, *p* = 6*e*−5 and for 500–700 ms window: *F*_(1, 68)_ = 37.85, *p* = 9*e*−10].

#### 3.2.2. Effect of Word Learning

**Figure 4:**
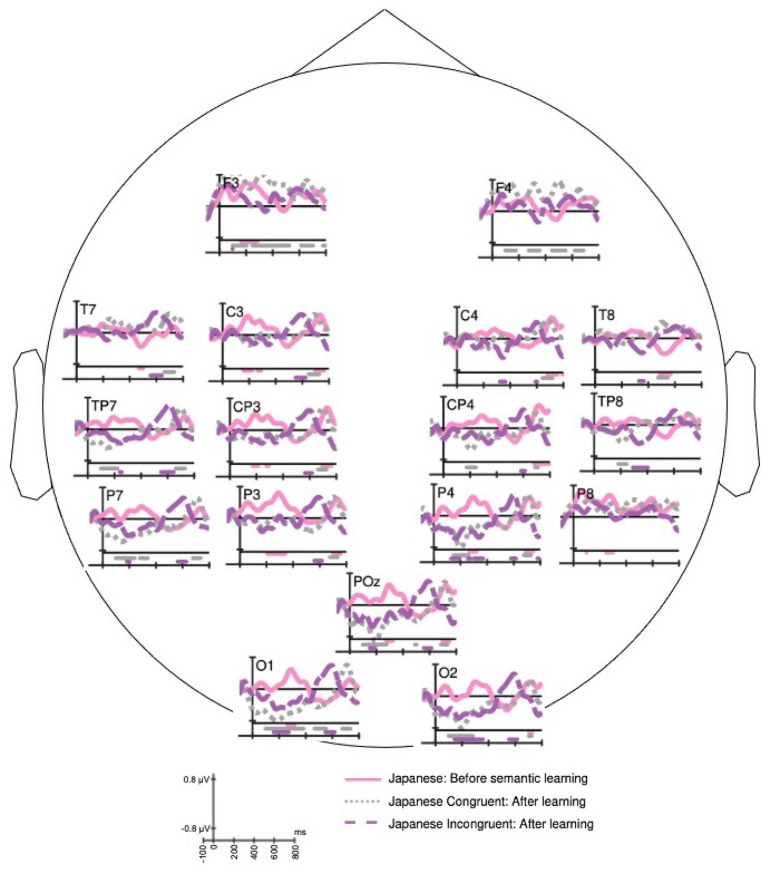
The grand average of event-related potential (ERP) responses to Japanese end-word before learning its meaning (solid), Japanese congruent end-word (dotted), and Japanese in-congruent end-word (dashed) after learning the meaning. The horizontal bars drawn in the bottom of each plot signify the time regions with significant (*t*-test with *p* < 0.05) ERP amplitude from the value of 0. The bars have the same color of the associated waveform.

Figure 4 shows the ERP response for the same set of Japanese language words before and after learning the semantics. The Japanese words exposed without semantic knowledge (solid line) evoke early positive peaks around 100 and 300 ms. This P300 response is possibly an indicator of exposure to novel information. The P100 and P300 responses disappear in the exposures after the subject learned the meaning of the word. The three responses in Figure 4 differ in the peak amplitude of N200 component. The ERP of Japanese end-word before semantic learning elicited a negative dip between P100 and P300 peaks. The ERP of Japanese congruent end-word after semantic learning elicited significant negative peak around 200 ms over parieto-occipital and occipital electrode sites. The incongruent end-word post semantic learning also elicited a negative peak, but with a lower peak amplitude than congruent case. The Japanese end-word response before semantic learning (solid line) and Japanese congruent response post semantic learning (dotted line) have a negative deflection before 600 ms. Both these responses also have LPC after 600 ms, which possibly is an indicator of the recognition of code-switch. In summary, the ERPs for congruent [*t*_(16)_ = 3.97, *p* = 5*e*−04] and incongruent [*t*_(16)_ = 5.89, *p* = 1.1*e*−05] conditions post semantic learning elicit significantly different responses in 500–700 ms from word onset.

**Figure 5:**
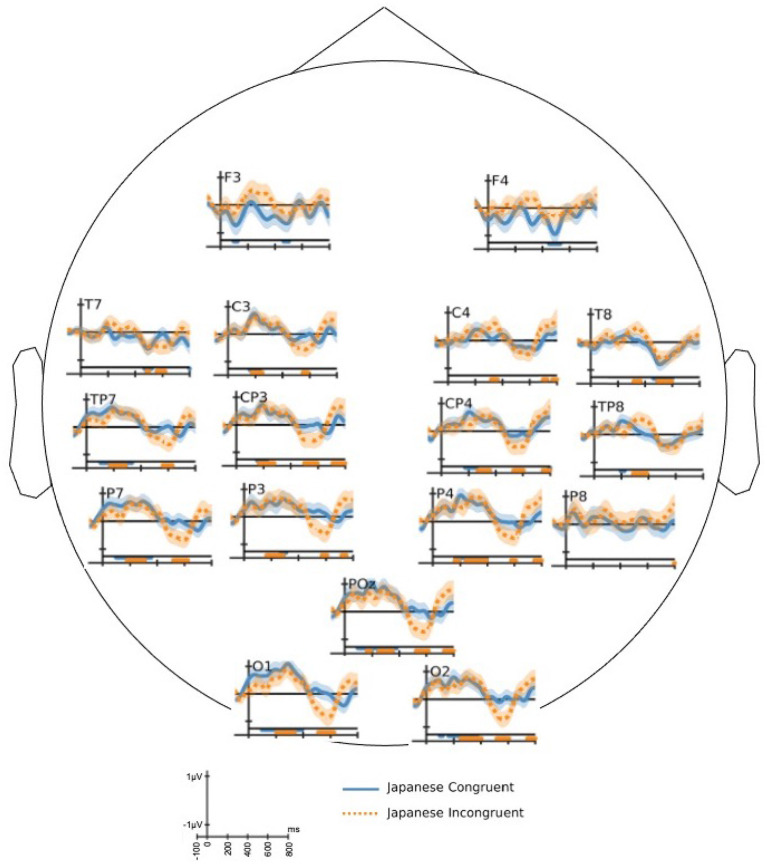
Grand average difference event-related potential (ERP) response of Japanese Congruent end-word from Japanese end-word response before learning its meaning (solid), and Japanese in-congruent end-word from Japanese end-word response before learning its meaning (dotted). The horizontal bars drawn in the bottom of each plot signify the time regions with significant (two-sample *t*-test with *p* < 0.05) difference in the ERP response. The bars have the same color of the associated difference waveform.

Figure 5 shows the differences in EEG responses to Japanese end-words before and after learning its meaning. The difference ERP wave forms of congruent (solid) and incongruent (dotted) conditions do not show any significant difference in 0–500 ms range. Both the conditions evoke significant negative peak around 200 ms. It is also noted that there is significant difference between before and after learning in the in-congruent condition than for congruent condition.

#### 3.2.3. Effect of Phonetic Similarity to Known Words

**Figure 6:**
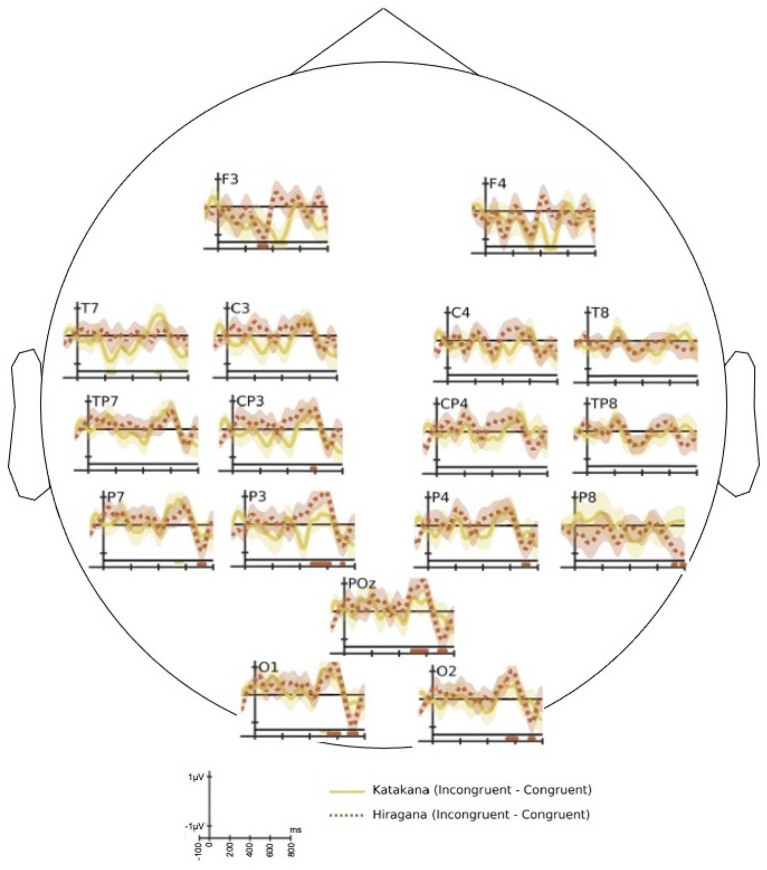
The grand average of difference event-related potential (ERP) of congruent end-word response from in-congruent end-word response for katakana words (solid) and hiragana words (dotted). The horizontal bars drawn in the bottom of each plot signify the time regions with significant (two-sample *t*-test with *p* < 0.05) difference in the ERP response. The bars have the same color of the associated difference waveform.

Figure 6 shows the difference ERP response for katakana (loan words in Japanese) and hiragana words separately. It shows the difference ERP of congruent condition from incongruent condition. Both hiragana and katakana words show significant P600 response. We performed a paired *t*-test over a cluster of centro-parietal and parietal electrode locations in the time-window 500–700 ms to ascertain the statistical significance. The difference ERP of katakana words showed *t*_(16)_ = 3.39, *p* = 1.87*e*−03 and that of hiragana words showed *t*_(16)_ = 4.18, *p* = 3.53*e*−04 in the P600 window. Thus, the hiragana words show larger P600 amplitude than katakana words [*t*_(16)_ = 3.91, *p* = 6.24*e*−04]. The statistical significance is more established for hiragana words in the occipital and left-parietal electrode locations.

### 3.3. Topographical Analysis

**Figure 7:**
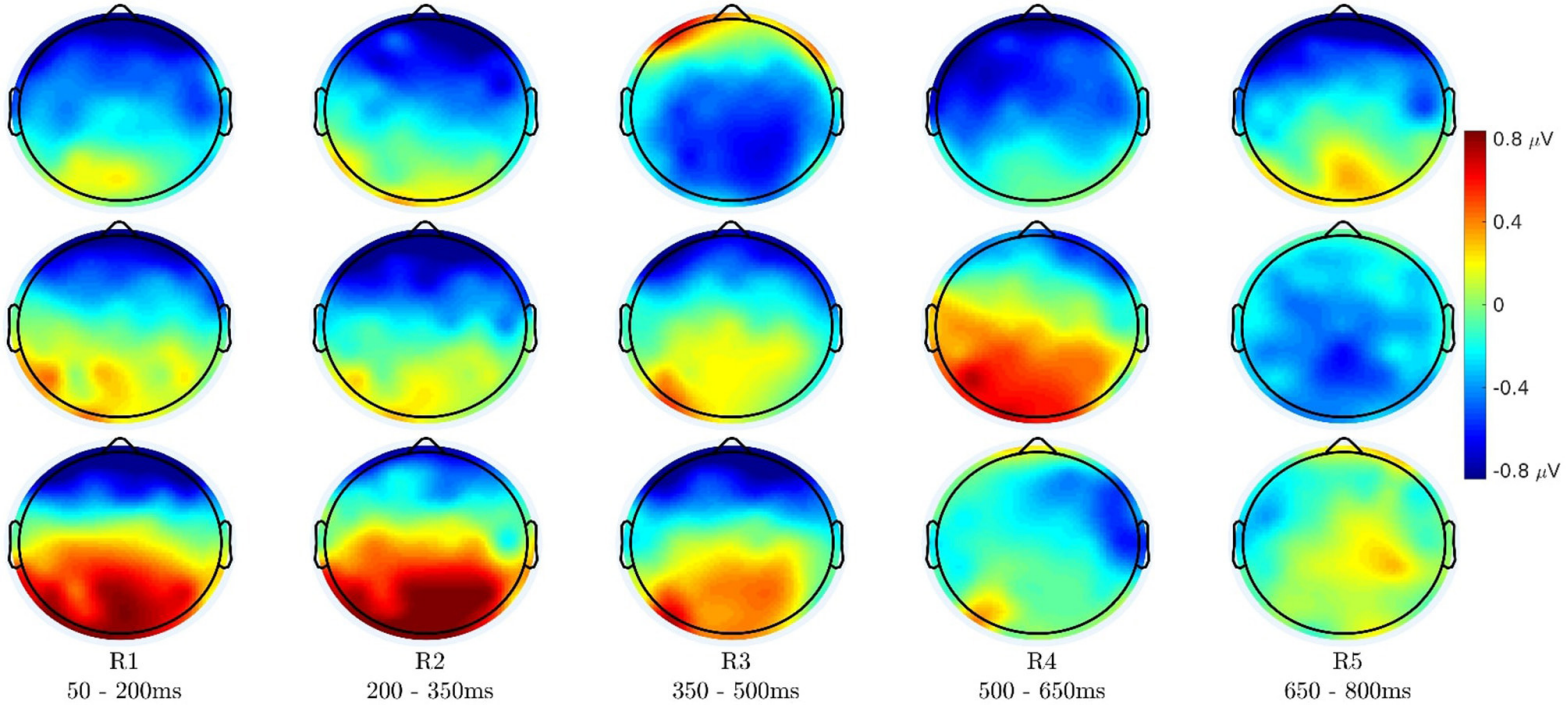
Topography distribution of mean difference of event-related potential (ERP) (grand-average) amplitudes in different time-windows. (Top) Difference of English congruent end-word responses from English incongruent end-word responses (S1). (Middle) Difference of Japanese congruent end-word responses from Japanese incongruent end-word responses (S3). (Bottom) Difference of Japanese end-word responses before learning its meaning from the Japanese end-word responses after learning its meaning (both in congruent context).

Figure 7 shows the topographical distribution of difference of ERP (grand-average) amplitudes in different time-windows. The mean value of ERP amplitudes in each time-window is plotted here. The top row shows the difference of English congruent end-word responses from English incongruent end-word responses, the middle row shows the difference of Japanese congruent end-word responses from Japanese incongruent end-word responses, and the bottom row shows the difference of Japanese end-word responses before learning its meaning from the Japanese end-word responses after learning its meaning.

As shown in previous works, the N400 response is significant over the centro-parietal region (see Figure 7 top row). Similarly, the Japanese congruent vs. incongruent difference is significant over the centro-parietal region in 450–650 ms time-window as seen in Figure 7 middle row. It is more evident in the left hemisphere than the right hemisphere of the scalp. The last row of Figure 7 shows ERP differences between the EEG responses before and after semantic learning in frontal, parietal, and occipital regions in the initial time-windows after the end-word onset. The response over the rear part of the brain is low in magnitude from 350 ms onwards, while the response in the frontal part sustains longer. In the frontal electrodes, the ERP after semantic learning is more positive than before semantic learning. The P100, N200, and P300 components contribute to the higher positivity over the parietal and occipital regions. This is also more pronounced in the left hemisphere than the right.

**Figure 8:**
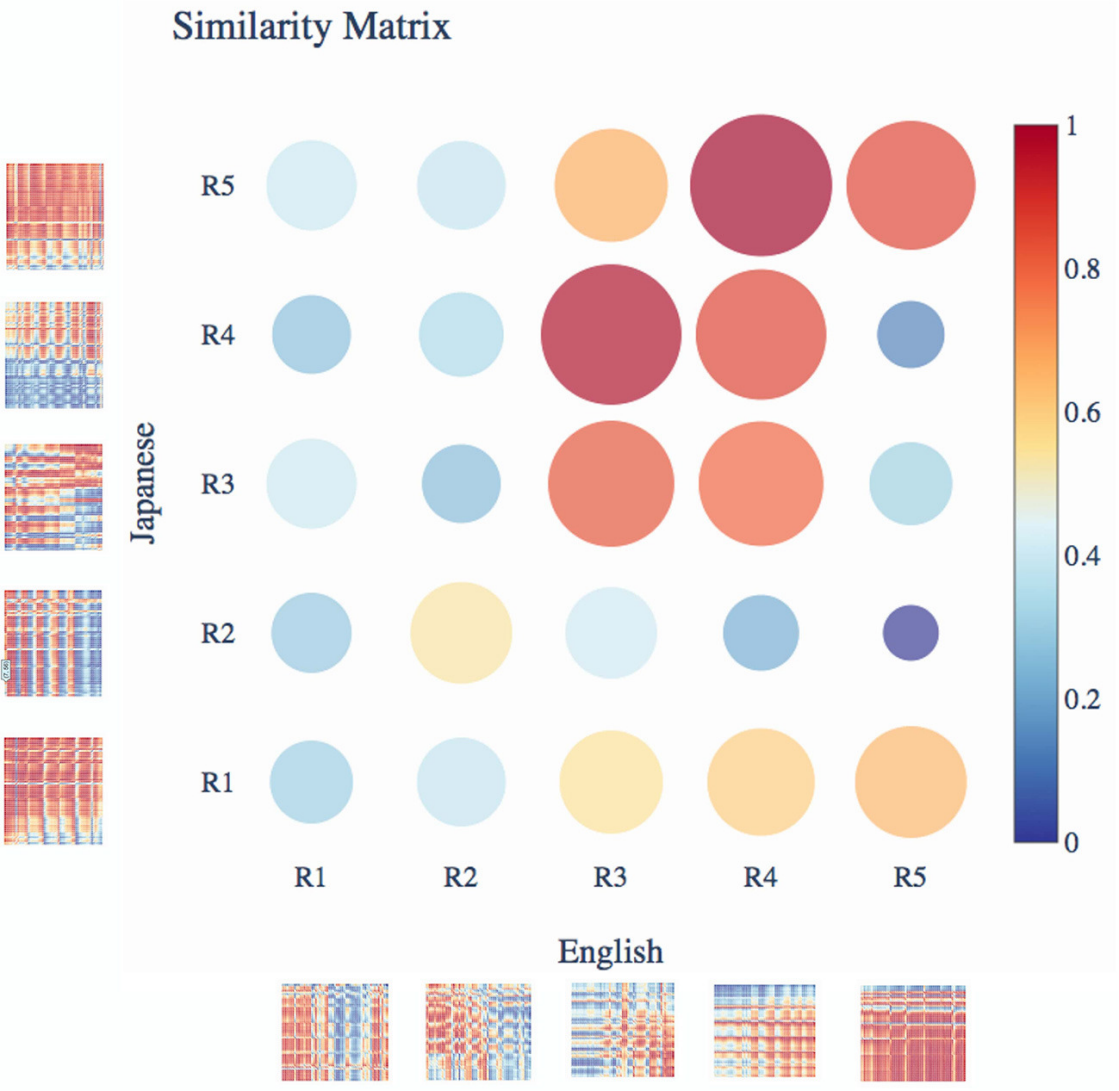
Distance matrix between English and Japanese correlation matrices in different time-windows. The cross-channel correlation is computed between the congruent and incongruent end-word event-related potential (ERP) responses across all channels for English (S1) and Japanese (S3). The time regions in the figure are as follows R1: 50–200 ms, R2: 200–350 ms, R3: 350–500 ms, R4: 500–650 ms, and R5: 650–800 ms. The highest similarity was between English responses at R4 and Japanese responses at R5.

The distance matrix shown in Figure 8 is computed between the congruent and incongruent end-word ERP responses across all channel pairs (more details of the correlation analysis are given in Supplementary Material, Section 3). It is computed for different time-windows as shown in Figure 8 to compare the correlation plot of the ERP waveforms for the two languages. The distance matrix in Figure 8 shows that the English difference response in R3 (350–500 ms) has high similarity (least distance) with Japanese difference response in R4 (500–650 ms). Similarly, we observe a high similarity between the difference response of English in R4 (500–650 ms) window with difference response of Japanese in R5 (650–800 ms) window.

### 3.4. Statistical Analysis of ERP Effects

We have used the mean amplitudes extracted from five non-overlapping time-windows of 150 ms duration between 50 and 800 ms from the word onset in repeated-measures ANOVA. The ANOVA used four within-subject factors: language (English/Japanese), congruency (congruent/incongruent), learning (before/after), and scalp region (frontal/central/parietal). The ERP effects suggested group difference at particular topographic regions. The language and congruency had a significant interaction in all time-windows except at 200–350 ms. It should be noted that the interaction is highly significant with a larger F-ratio in the 350–500 ms window (*F* = 31.09, *p* < 0.01) and 500–650 ms window (*F* = 73.08, *p* < 0.01). The language and scalp region factors had significant interaction in two time-windows: at 50–200 and 500–650 ms. The factors of learning and scalp region had significant interactions in all time-windows except 500–650 ms. This implies that the process of learning had topographic selectivity. The highest significance is observed in the 200–350 ms window (*F* = 28.49, *p* < 0.01). The congruency and scalp region also had significant interactions in the 50–200 and 500–650 ms windows.

## Discussion

The grand average difference of ERP response of Japanese congruent end-word from incongruent end-word showed a P600 component (Figure 3). This can be attributed to semantic P600 component. The work by Kuperberg et al. (2003) showed that the violation of semantic congruity in sentences with strong semantic relationship between its noun and verb can evoke a semantic P600 response. It is also worth noticing that the congruent and incongruent semantic conditions showed different brain responses for familiar language at around 400 ms and for newly acquired words at around 600 ms. The semantic differences in Japanese words evoked a later response than the English words. This may be due to the reason that the newly learned words required a reanalysis to integrate itself with the sentence. The ERP of incongruent words evoked a significant positive peak while the ERP of congruent words evoked a negative peak around 600 ms (Figure 4).

Figure 6 shows that both katakana and hiragana words evoked P600 component in the difference ERP. The hiragana P600 response has higher amplitude than the katakana response over the parietal and parieto-occipital electrodes. The katakana words are loan words from English, but they are pronounced with the Japanese adaptation. As shown in the behavioral responses, human subjects find it easier to recall the meaning of katakana words and hence, the involved reanalysis is not as strong as the hiragana words. The observation that hiragana words have higher P600 amplitude than katakana words is similar to the higher amplitude of N400 observed for highly unexpected end-word in the known language (Brown and Hagoort, 1993; Curran et al., 1993; Holcomb, 1993; DeLong et al., 2005).

Figures 4, 5 show the effects of semantic learning. The Japanese words in the first exposure elicited a significant P300 response owing to the novelty of the stimuli. After semantic learning, the ERP of congruent and incongruent conditions did not have significant differences in the early part of the response. The differences are significant after 500 ms from the onset of the end-word. This shows that the semantic processing of the newly acquired words may occur with a delay of more than 500 ms from the word onset.

The foreign word at the end of the sentence is comprehended as a form change, since it evokes a P600 potential instead of N400 potential. This is comparable to the semantic illusion condition shown by Brouwer et al. (2012). The code-switching to a newly learned word at the end of the sentence requires re-analysis to integrate it with the prior sentence context. As shown in other code-switching studies like Van Hell et al. (2018) and Fernandez et al. (2019), we observe that P600 is elicited for the congruent end-word in context. In this work, we show that P600 potential is evoked while perceiving newly acquired word from a foreign language employed in a code-switched manner. Further, we see a difference in the P600 latency in the response for the newly acquired end-word used in congruent and incongruent context. When the newly acquired word is used in congruent condition, we see the positive peak appearing at a slightly later time with a comparable amplitude. This difference in the response to the Japanese word used in congruent and incongruent condition shows the ERP effects of rapid semantic acquisition of foreign language words. This difference in responses for congruent and incongruent cases for the newly acquired word illustrates that the ambiguity is recognized by involving a higher cognitive load. For the newly acquired words, the word used in congruent condition evokes a lesser positive potential around 600 ms from the word onset and shows a more positive deflection in 700–800 ms (peaking around 750 ms). This can be observed in Figure 4. This implies that the semantic integration of newly learned words occurs much later in time compared to the similar process in a proficient language word. The underlying cognitive process may also be bi-phasic: recognition and then integration of the meaning with the sentence context.

The topographic plots show that P600 responses are also stronger over the centro-parietal and parietal regions like the N400 response. But the P600 response has a left hemisphere selectivity. The scalp distributions of difference ERP before and after semantic learning elicit significant responses in the early part of the ERP waveform. It has strong positive response over the parietal and parieto-occipital regions owing to the differences in N200 response and positive response in the frontal part owing to the P300 response. The correlation analysis in Figure 8 shows that the highly negative correlation that exists between the EEG responses for congruent and incongruent conditions for English at about 400 ms also appear for Japanese stimuli but at a later time instant of 600 ms.

## Data Availability Statement

The raw data supporting the conclusions of this article will be made available by the authors, without undue reservation.

## Ethics Statement

The studies involving human participants were reviewed and approved by Institute Human Ethics Committee (IHEC), Indian Institute of Science, Bangalore. The patients/participants provided their written informed consent to participate in this study.

## Author Contributions

AS: conceptualization of this study, methodology, investigation, software, formal analysis, and writing-original draft and editing. PR: methodology and investigation. SG: conceptualization of this study, methodology, writing-review, supervision, and funding acquisition. All authors contributed to the article and approved the submitted version.

## Funding

This work was partly funded by the Ministry of Human Resource Development, Government of India, Department of Atomic Energy, Government of India (34/20/12/2018-BRNS/3408), the Pratiksha Trust and Department of Science and Technology, Government of India (DST-ECR/2017/01341).

## Conflict of Interest

The authors declare that the research was conducted in the absence of any commercial or financial relationships that could be construed as a potential conflict of interest.

## Publisher's Note

All claims expressed in this article are solely those of the authors and do not necessarily represent those of their affiliated organizations, or those of the publisher, the editors and the reviewers. Any product that may be evaluated in this article, or claim that may be made by its manufacturer, is not guaranteed or endorsed by the publisher.

## Footnotes

^1^ https://github.com/iiscleap/Semantics-EEG-ERPStudy

^2^ https://github.com/iiscleap/Semantics-EEG-ERPStudy

^3^ https://www.oxfordonlineenglish.com/english-level-test/listening

## References

[B1] Batterink L. Neville H. (2011). Implicit and explicit mechanisms of word learning in a narrative context: an event-related potential study. J. Cogn. Neurosci. 23, 3181–3196. 10.1162/jocn_a_00013 21452941 PMC3129368

[B2] Block C. K. Baldwin C. L. (2010). Cloze probability and completion norms for 498 sentences: behavioral and neural validation using event-related potentials. Behav. Res. Methods 42, 665–670. 10.3758/BRM.42.3.665 20805588

[B3] Borovsky A. Elman J. L. Kutas M. (2012). Once is enough: N400 indexes semantic integration of novel word meanings from a single exposure in context. Lang. Learn. Dev. 8, 278–302. 10.1080/15475441.2011.614893 23125559 PMC3484686

[B4] Borovsky A. Kutas M. Elman J. (2010). Learning to use words: Event-related potentials index single-shot contextual word learning. Cognition 116, 289–296. 10.1016/j.cognition.2010.05.004 20621846 PMC2904319

[B5] Brouwer H. Fitz H. Hoeks J. (2012). Getting real about semantic illusions: rethinking the functional role of the p600 in language comprehension. Brain Res. 1446, 127–143. 10.1016/j.brainres.2012.01.055 22361114

[B6] Brown C. Hagoort P. (1993). The processing nature of the N400: evidence from masked priming. J. Cogn. Neurosci. 5, 34–44. 10.1162/jocn.1993.5.1.34 23972118

[B7] Brysbaert M (2019). How many participants do we have to include in properly powered experiments? A tutorial of power analysis with reference tables. J. Cogn. 2:16. 10.5334/joc.72 31517234 PMC6640316

[B8] Chikamatsu N. Yokoyama S. Nozaki H. Long E. Fukuda S. (2000). A Japanese logographic character frequency list for cognitive science research. Behav. Res. Methods Instrum. Comput. 32, 482–500. 10.3758/BF03200819 11029823

[B9] Clark E. V (1973). “What's in a word? On the child's acquisition of semantics in his first language,” in Cognitive Development and Acquisition of Language (Elsevier), 65–110. 10.1016/B978-0-12-505850-6.50009-8

[B10] Comerchero M. D. Polich J. (1999). P3A and P3B from typical auditory and visual stimuli. Clin. Neurophysiol. 110, 24–30. 10.1016/S0168-5597(98)00033-1 10348317

[B11] Coulson S. King J. W. Kutas M. (1998). Expect the unexpected: Event-related brain response to morphosyntactic violations. Lang. Cogn. Process. 13, 21–58. 10.1080/016909698386582

[B12] Curran T. Tucker D. M. Kutas M. Posner M. I. (1993). Topography of the N400: brain electrical activity reflecting semantic expectancy. Electroencephalogr. Clin. Neurophysiol. 88, 188–209. 10.1016/0168-5597(93)90004-9 7684968

[B13] DeLong K. A. Urbach T. P. Kutas M. (2005). Probabilistic word pre-activation during language comprehension inferred from electrical brain activity. Nat. Neurosci. 8:1117. 10.1038/nn1504 16007080

[B14] Delorme A. Makeig S. (2004). EEGLAB: an open source toolbox for analysis of single-trial EEG dynamics including independent component analysis. J. Neurosci. Methods 134, 9–21. 10.1016/j.jneumeth.2003.10.009 15102499

[B15] Dikker S. Pylkkanen L. (2011). Before the N400: effects of lexical-semantic violations in visual cortex. Brain Lang. 118, 23–28. 10.1016/j.bandl.2011.02.006 21458057

[B16] Faul F. Erdfelder E. Lang A.-G. Buchner A. (2007). G* power 3: a flexible statistical power analysis program for the social, behavioral, and biomedical sciences. Behav. Res. Methods 39, 175–191. 10.3758/BF03193146 17695343

[B17] Federmeier K. D. Kutas M. (1999a). Right words and left words: electrophysiological evidence for hemispheric differences in meaning processing. Cogn. Brain Res. 8, 373–392. 10.1016/S0926-6410(99)00036-1 10556614

[B18] Federmeier K. D. Kutas M. (1999b). A rose by any other name: long-term memory structure and sentence processing. J. Mem. Lang. 41, 469–495. 10.1006/jmla.1999.2660

[B19] Fernandez C. B. Litcofsky K. A. van Hell J. G. (2019). Neural correlates of intra-sentential code-switching in the auditory modality. J. Neurolinguist. 51, 17–41. 10.1016/j.jneuroling.2018.10.004

[B20] Friederici A. D (1995). The time course of syntactic activation during language processing: a model based on neuropsychological and neurophysiological data. Brain Lang. 50, 259–281. 10.1006/brln.1995.1048 7583190

[B21] Friederici A. D (2002). Towards a neural basis of auditory sentence processing. Trends Cogn. Sci. 6, 78–84. 10.1016/S1364-6613(00)01839-8 15866191

[B22] Hagoort P. Brown C. Groothusen J. (1993). The syntactic positive shift (SPS) as an erp measure of syntactic processing. Lang. Cogn. Process. 8, 439–483. 10.1080/01690969308407585

[B23] Hagoort P. Brown C. M. (2000). ERP effects of listening to speech: semantic ERP effects. Neuropsychologia 38, 1518–1530. 10.1016/S0028-3932(00)00052-X 10906377

[B24] Hoeks J. C. Stowe L. A. Doedens G. (2004). Seeing words in context: the interaction of lexical and sentence level information during reading. Cogn. Brain Res. 19, 59–73. 10.1016/j.cogbrainres.2003.10.022 14972359

[B25] Holcomb P. J (1993). Semantic priming and stimulus degradation: implications for the role of the N400 in language processing. Psychophysiology 30, 47–61. 10.1111/j.1469-8986.1993.tb03204.x 8416062

[B26] Kaan E. Harris A. Gibson E. Holcomb P. (2000). The P600 as an index of syntactic integration difficulty. Lang. Cogn. Process. 15, 159–201. 10.1080/01690960038608415722211

[B27] Kuperberg G. R. Sitnikova T. Caplan D. Holcomb P. J. (2003). Electrophysiological distinctions in processing conceptual relationships within simple sentences. Cogn. Brain Res. 17, 117–129. 10.1016/S0926-6410(03)00086-7 12763198

[B28] Kutas M. Federmeier K. D. (2011). Thirty years and counting: finding meaning in the N400 component of the event-related brain potential (ERP). Annu. Rev. Psychol. 62, 621–647. 10.1146/annurev.psych.093008.131123 20809790 PMC4052444

[B29] Kutas M. Hillyard S. (1980). Reading senseless sentences: brain potentials reflect semantic incongruity. Science 207, 203–205. 10.1126/science.7350657 7350657

[B30] Kutas M. Hillyard S. A. (1983). Event-related brain potentials to grammatical errors and semantic anomalies. Mem. Cogn. 11, 539–550. 10.3758/BF03196991 6656613

[B31] Kutas M. Hillyard S. A. (1984). Brain potentials during reading reflect word expectancy and semantic association. Nature 307:161. 10.1038/307161a0 6690995

[B32] Kutas M. Neville H. J. Holcomb P. J. (1987). A preliminary comparison of the N400 response to semantic anomalies during reading, listening and signing. Electroencephalogr. Clin. Neurophysiol. Suppl. 39, 325–330. 3477442

[B33] Leckey M. Federmeier K. D. (2020). The P3b and P600 (s): positive contributions to language comprehension. Psychophysiology 57:e13351. 10.1111/psyp.13351 30802979 PMC7934419

[B34] Luck S. J. Vogel E. K. Shapiro K. L. (1996). Word meanings can be accessed but not reported during the attentional blink. Nature 383, 616–618. 10.1038/383616a0 8857535

[B35] McCallum W. Farmer S. Pocock P. (1984). The effects of physical and semantic incongruites on auditory event-related potentials. Electroencephalogr. Clin. Neurophysiol. 59, 477–488. 10.1016/0168-5597(84)90006-6 6209114

[B36] Mestres-Missé A. Rodriguez-Fornells A. Münte T. F. (2007). Watching the brain during meaning acquisition. Cereb. Cortex 17, 1858–1866. 10.1093/cercor/bhl094 17056648

[B37] Moreno E. M. Federmeier K. D. Kutas M. (2002). Switching languages, switching palabras (words): an electrophysiological study of code switching. Brain Lang. 80, 188–207. 10.1006/brln.2001.2588 11827443

[B38] Moreno E. M. Rodríguez-Fornells A. Laine M. (2008). Event-related potentials (ERPs) in the study of bilingual language processing. J. Neurolinguist. 21, 477–508. 10.1016/j.jneuroling.2008.01.003

[B39] Nigam A. Hoffman J. E. Simons R. F. (1992). N400 to semantically anomalous pictures and words. J. Cogn. Neurosci. 4, 15–22. 10.1162/jocn.1992.4.1.15 23967854

[B40] Nobre A. C. Mccarthy G. (1995). Language-related field potentials in the anterior-medial temporal lobe: II. Effects of word type and semantic priming. J. Neurosci. 15, 1090–1098. 10.1523/JNEUROSCI.15-02-01090.1995 7869085 PMC6577813

[B41] Olah B (2007). English loanwords in Japanese: effects, attitudes and usage as a means of improving spoken english ability. Bunkyo Gakuin Daigaku Ningen Gakubu Kenkyū Kiyo 9, 177–188.

[B42] Osterhout L. Holcomb P. J. (1992). Event-related brain potentials elicited by syntactic anomaly. J. Mem. Lang. 31, 785–806. 10.1016/0749-596X(92)90039-Z

[B43] Perfetti C. A. Wlotko E. W. Hart L. A. (2005). Word learning and individual differences in word learning reflected in event-related potentials. J. Exp. Psychol. 31:1281. 10.1037/0278-7393.31.6.1281 16393047

[B44] Polich J (2007). Updating P300: an integrative theory of P3a and P3b. Clin. Neurophysiol. 118, 2128–2148. 10.1016/j.clinph.2007.04.019 17573239 PMC2715154

[B45] Soman A. Madhavan C. Sarkar K. Ganapathy S. (2019). An EEG study on the brain representations in language learning. Biomed. Phys. Eng. Exp. 5, 25–41. 10.1088/2057-1976/ab0243

[B46] Speidel G. E. Nelson K. E. (2012). The Many Faces of Imitation in Language Learning, Vol. 24. Springer Science & Business Media.

[B47] Szűcs D. Soltész F. Czigler I. Csépe V. (2007). Electroencephalography effects to semantic and non-semantic mismatch in properties of visually presented single-characters: the N2b and the N400. Neurosci. Lett. 412, 18–23. 10.1016/j.neulet.2006.08.090 17141414

[B48] Tanner D. Grey S. van Hell J. G. (2017). Dissociating retrieval interference and reanalysis in the P600 during sentence comprehension. Psychophysiology 54, 248–259. 10.1111/psyp.12788 27859315

[B49] Van Hell J. G. Fernandez C. B. Kootstra G. J. Litcofsky K. A. Ting C. Y. (2018). Electrophysiological and experimental-behavioral approaches to the study of intra-sentential code-switching. Linguist. Approach. Biling. 8, 134–161. 10.1075/lab.16010.van

[B50] Van Petten C. Kutas M. (1990). Interactions between sentence context and word frequency in event-related brain potentials. Mem. Cogn. 18, 380–393. 10.3758/BF03197127 2381317

[B51] Wlotko E. W. Lee C.-L. Federmeier K. D. (2010). Language of the aging brain: event-related potential studies of comprehension in older adults. Lang. Linguist. Compass 4, 623–638. 10.1111/j.1749-818X.2010.00224.x 20823949 PMC2930790

[B52] Zwaan R. A. Pecher D. Paolacci G. Bouwmeester S. Verkoeijen P. Dijkstra K.. (2018). Participant nonnaiveté and the reproducibility of cognitive psychology. Psychon. Bull. Rev. 25, 1968–1972. 10.3758/s13423-017-1348-y 28744765

