# Supplementary material for: ERP Evidences of Rapid Semantic Learning in Foreign Language Word Comprehension
Source: Front Neurosci. 2022 Mar 3;16:763324. doi: 10.3389/fnins.2022.763324 (PMC8927935; doi:10.3389/fnins.2022.763324)
Supplement: Supplementary file 1 [file Data_Sheet_1.pdf]

Supplementary Material

Table S1. Experimental Details

Particulars	Details
Presentation Software	Python based GUI developed by the authors
Monitor	Samsung 27"
Loudspeaker	Dell Ax210 USB Stereo
Microphone	CAD u37

1 STIMULI SET

Table S2: List of highly predictable English sentences and end words in different conditions used in the experiment.

Sentences and End words				
Sentence Prefix	English congruent end word	English incongruent	Japanese congruent	Japanese incongruent
She made the bed with clean	bedsheets	swan	mokkori	maggu
My T.V has a fifty-inch	screen	van	gamen	komugi
The beer drinkers raised their	mugs	hen	maggu	mokkori
Harry could see the blooming	flowers	rope	hanabana	senro
The bread was made from whole	wheat	garage	komugi	hon
I made a phone call from a	booth	bowl	būsu	chizu
A termite looks like an	ant	goalpost	ari	kēki
For your birthday I baked a	cake	map	kēki	ami
Kevin went to the library to read	books	calf	hon	kouzui
The fruit was shipped in wooden	box	tablets	hako	hachinosu
We're lost so let's look at the	map	spoon	mappu	ari
Household goods are moved in a	van	beehive	kasha	naifu
The honey bees swarmed round the	beehive	thorns	hachinosu	besuto
I cut my finger with a	knife	screen	naifu	nedoko
The candle flame melted the	wax	clock	rou	toge
This key won't fit in the	lock	pool	jou	suwan
The baby slept in the	crib	lock	nedoko	rou
A rose bush has prickly	thorns	pie	toge	koromo
Ruth poured the water down the	sink	ant	nagashi	genkotsu
The cop wore a bullet-proof	vest	jar	besuto	taiko
After his bath he wore a	robe	bomb	koromo	shippu
The soup was served in a	bowl	cot	bouru	kesshouten
They marched to the beat of the	drum	cloth	doramu	nuno
The sailor cleaned the deck of the	ship	broom	senpaku	nawa
They played a game of cat and	mouse	truck	nezumi	torakku
We shipped the furniture by	truck	booth	torakku	supūn
He tossed the drowning man a	rope	crib	rōpu	suwan
Stir your coffee with a	spoon	mouse	supūn	kuraun
At breakfast he drank apple	juice	gown	jūsu	bouru
He hit me with a clenched	fist	police	genkotsu	oin
The king wore a golden	crown	drum	kuraun	houki
The duck swam with the white	swan	ant	suwan	shako
Let's decide by tossing a	coin	crown	koin	būsu
The girl swept the floor with a	broom	truck	houki	tokei
We heard the ticking of the	clock	mugs	tokei	yakuzai
The doctor prescribed the	tablets	sling	yakuzai	medaru
Unlock the door and turn the	knob	flowers	nobu	sankakukin
Her entry should win a	medal	lock	medaru	torappu
The mouse was caught in the	trap	ship	torappu	nedoko

Continuation of Table S2				
Sentence Prefix	English congruent end word	English incongruent	Japanese congruent	Japanese incongruent
The house was robbed by a	thief	mop	dorobou	benchi
Wash the floor with a	mop	coin	moppu	tento
Harry slept on the folding	cot	medal	nedoko	mendori
The man is sitting on the	bench	mold	benchi	kouzui
The heavy rains caused a	flood	bench	kouzui	kaeru
The chicks followed the mother	hen	mat	mendori	ushi
We camped out in our	tent	juice	tento	hitsuji
The pond was full of croaking	frogs	wheels	kaeru	hato
The shepherd watched his flock of	sheep	fist	hitsuji	mune
The swimmer dove into the	pool	cap	pu-ru	gaun
The cigarette smoke filled his	lungs	bread	mune	ryourin
The bride wore a white	gown	wheat	koromo	koushi
We swam at the beach during high	tide	trap	shio	satsu
A bicycle has two	wheels	bedsheets	ryourin	kyappu
The cow gave birth to a	calf	tent	koushi	pai
Paul was arrested by the	police	track	satsu	matto
On a sunny day she wore a	cap	dove	kyappu	jūsu
For dessert he had apple	pie	knob	pai	dorobou
Please wipe your feet on the	mat	ox	matto	nobu
When she got out of the car she closed the	Door	stars	doa	sakana
He mailed the letter without a	Stamp	milk	kitte	miruku
In the shower he washed his face with	Soap	plates	sekken	kyoukai
After every meal it’s good to brush your	Teeth	gifts	hanarabi	isu
He brought his bait to the lake to catch	Fish	pants	sakana	cha
Joan fed her baby some warm	Milk	eyes	miruku	taiyou
Every Sunday the family goes to	Church	bank	kyoukai	shita
The man happily sat down in the comfortable	Chair	socks	isu	ushi
He liked lemon and sugar in his	Tea	shark	cha	shuzu
The player’s cap protected him from the	Sun	nest	taiyou	yubiwa
While eating Steve accidentally bit his	Tongue	fire	shita	kagi
The farmer spend the morning milking his	Cow	yarn	ushi	hikouki
Susan put on the socks and	Shoes	bag	kutsu	jumoku
Bob proposed and gave her a diamond	Ring	kite	yubiwa	hoshiboshi
Carolyn couldn’t start her car without the right	Keys	door	kagi	
Tim joined the Airforce as he always wanted to fly an	Aeroplane	stamp	hikouki	kaban
To learn about their ancestors they drew a family	Tree	fish	jumoku	sokkusu
In the night sky it is easier to see all the	Stars	church	hoshiboshi	omeme
It was windy enough to fly a	Kite	tea	kaito	ginkou
The thief ran by and snatched the lady’s	Bag	tongue	kaban	doa
Derek’s feet were cold, so he put on some	Socks	chair	sokkusu	kouhan
Without her sunglasses the sun hurt Annie’s	Eyes	cow	omeme	zubon
He deposited his new paycheck at the	Bank	ring	ginkou	sekken
On her birthday she excitedly opened the	Gifts	shoes	purezento	nesuto
After dinner the maid collected the family’s	Plates	sun	kouhan	kaji
Sid needed a belt to hold up his	Pants	fish	zubon	neiru
The birds lay the eggs in the	Nest	teeth	nesuto	purezentsu
Dan gathered more wood for the	Fire	aeroplane	kaji	kitte
We had a candle-light dinner in a lakeview	restaurant	camera	restoran	pasupoto
Mary missed her company bus, so she took a	taxi	passport	takushi	restoran
She clicked the picture with a	camera	taxi	kamera	takushi
Travelling to foreign land needs a visa and	passport	restaurant	pasupoto	kamera

2 EXPERIMENTAL PARADIGM

The audio files of the above stimuli set is available at https://github.com/iiscleap/eeg_semantics_stimuli. Both English sentences and Japanese words were recorded by a multilingual speaker proficient in both languages and hence, we eliminated speaker change bias. The audio files were recorded in a noise proof sound booth with a CAD u37 microphone at a sampling rate of 44.1kHz.

The experimental paradigm of the different phases of the experiment are graphically described below.

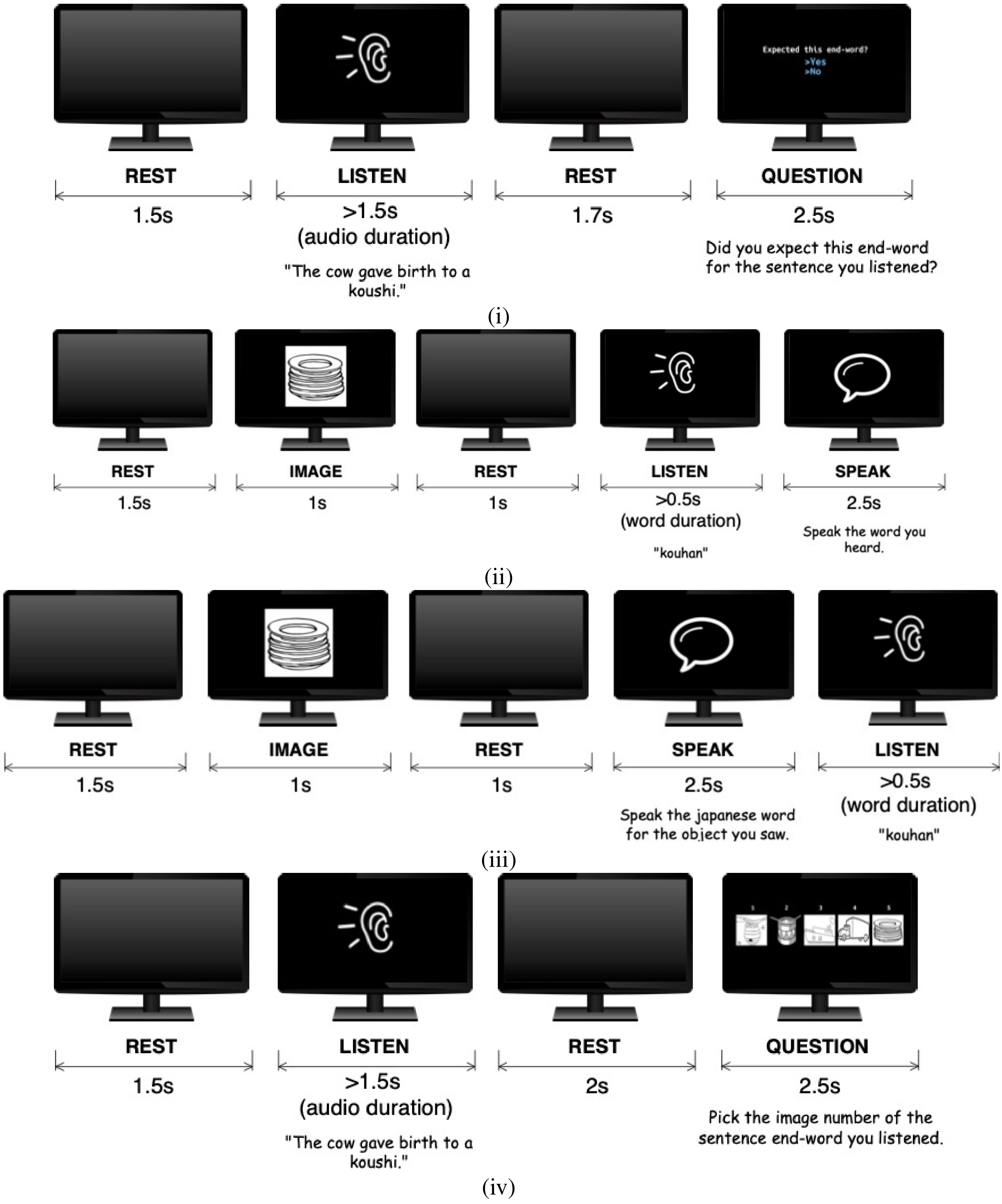

Figure S1. The different stages of the experiment is shown pictorially.(i) Listening Session, (ii) Learning Session, (iii) Recall Session, and (iv) Test Session. These stages are in serial order for a particular word. But as a whole, stages for different words are interspersed and randomized. (Sources of Images used in the experiment: ©*pixabay.com, unsplash.com, pexels.com*)

Stimuli Condition	Duration of End-word [min. - max.] (in s)
English Congruent	[0.2 - 1.1]
English Incongruent	[0.3 - 1.3]
Japanese Congruent	[0.4 - 1.3]
Japanese Incongruent	[0.4 - 1.3]

Table S3. The table shows the range of duration of end-word of sentences in different stimuli conditions. Note: We used the same set of words for different conditions for a language. But the duration can vary slightly as it is spoken as part of a sentence in different instances.

3 KATAKANA AND HIRAGANA WORDS

Hiragana and katakana — collectively referred to by the generic term kanamoji — are both syllabic alphabets of 47 characters, each of which represents a sound. Some of the characters between the two alphabets even represent the exact same sounds and look quite similar to one another. Hiragana (phonetic sounds) are basically used for particles, words and parts of words. Katakana (phonetic sounds) are basically used for foreign/loan words.

Figure S2 shows that event related potential evoked by katakana and hiragana words before semantic exposure does not show any significant difference. Note that, a statistical significance would be highlighted as black horizontal bar in the figures. The absence of any horizontal bars means that, for all the electrodes considered here, the ERP responses for katakana and hiragana words were not statistically significantly different.

4 NUMBER OF SUBJECTS AND NUMBER OF TRIALS

4.1 Regarding number of subjects:

The number of participants in similar studies published in literature were in the range of 15-20 participants. For example, Coulson and Wu (2005), Kutas and Federmeier (2000), Bae and Luck (2018), Schendan and Kutas (2002), Meade et al. (2018), and Kumar et al. (2021). Furthermore, Picton et al. (2000), committee report published in Society for Psychophysiological Research, suggests that the criterion for deciding the number of subjects for an ERP experiment as: “the number of subjects in an experiment must be sufficient to allow statistical tests to demonstrate the experimental effects and to support generalization of the results.” We have employed appropriate t-tests and ANOVA tests to ensure the statistical validity of the results reported. Each result in the paper is supported along with it’s obtained statistical power.

4.2 Regarding Multiple Trials:

Every Japanese word was presented the same number of times. The number of exposures for each word is 5. The first one is during session 1 in sentence context (S1), followed by 2 exposures in isolated fashion during session 2 (S2) [learning phase] and then two exposures in S3, where the word was introduced again with a sentence context (congruent and incongruent). Each of the sessions (S1, S2, and S3) for the 90 Japanese words (and the English words used in S1) consisted of about 20 minutes of recording duration. Thus, the three sessions were recorded in an interleaved fashion in one recording setup for each of the subject. Thus, the subject did not get exposed more to katakana words than hiragana words.

5 CORRELATION PLOT

Let the ERP waveform for channel c for language l and for condition s be denoted as $x_c^{s,l}(t)$ where channel c ranges from $1-64$, language l corresponds to 1 for English and 2 for Japanese, condition s corresponds to 1 for congruent and 2 for incongruent condition. For different time regions R1-R5 (where R1 ranges from $50-200$ms, R2 from $200-350$ms, R3 from $350-500$ms, R4 from $500-650$ms and R5 from $650-800$ms), the correlation matrix for each language l are computed using,

$$\mathbf{C}^l_{R_j}(i,k) = \sum_{t=s_{R_j}}^{e_{R_j}} x_i^{l,1}(t) x_k^{l,2}(t)$$

where R_j corresponds to regions R1-R5 and s_{R_j} and e_{R_j} denote the start and end time instants of the region. Thus, the matrix of values $C^l_{R_j}$ denotes the cross correlation between the ERP responses for the

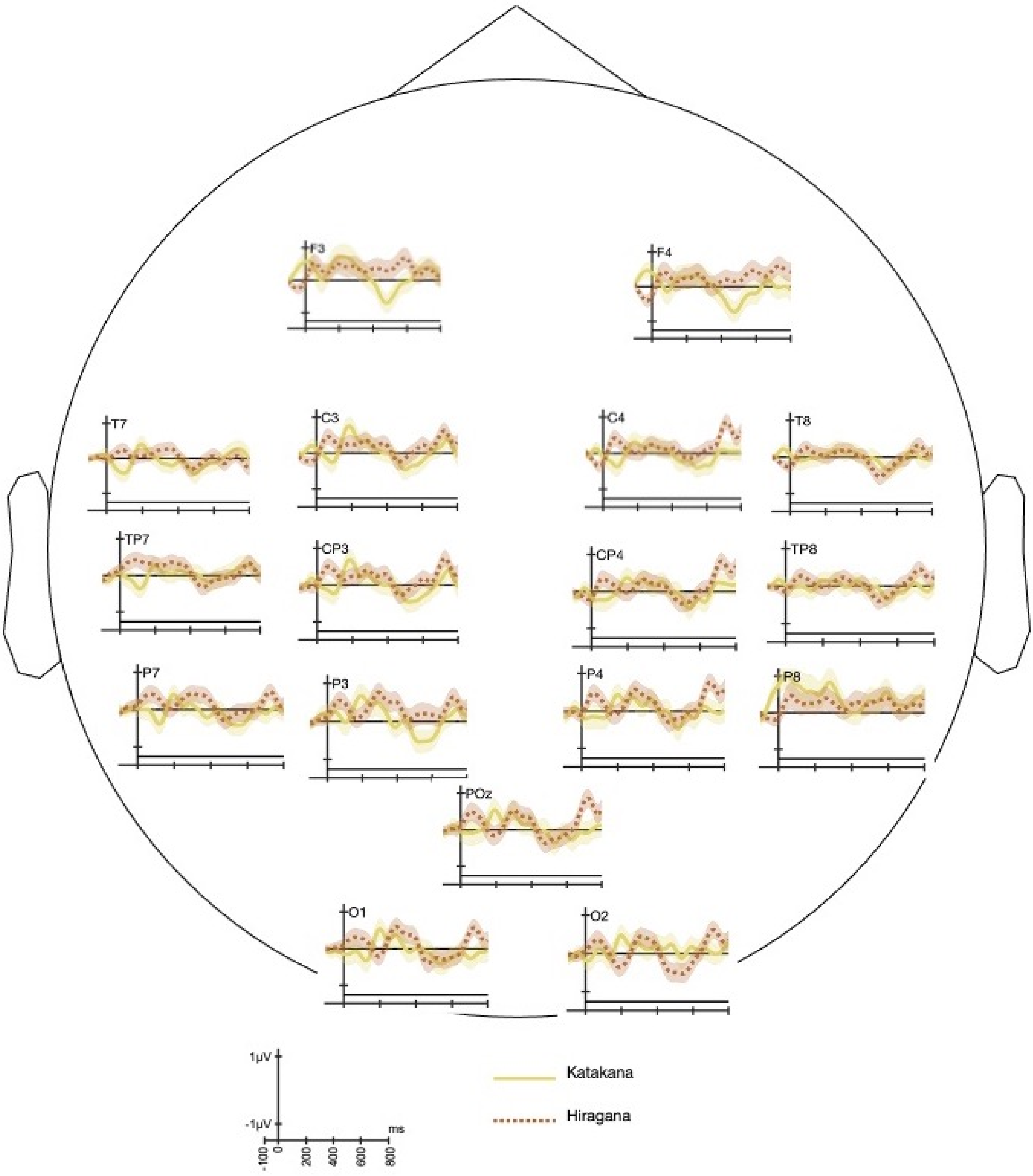

Figure S2. ERP plot for Katakana and Hiragana words in session 1 (before semantic exposure) of the experiment. Before knowing the meaning of the word, perception of both types of words did not show any significant difference in event related potentials. Two sample t-tests conducted did not give any time region with significant difference between the responses for two types of words (Note: If there is any significant region, that will be marked with horizontal bars below the black horizontal line at the bottom of each subplot in the figure).

congruent and incongruent condition. A high value at location (i, k) for this matrix indicates that for the channel pairs (i, k) the EEG responses to congruent and incongruent conditions are highly correlated (and time synchronized). Similarly, a low negative value indicates that for the channel pairs (i, k) the EEG responses to congruent and incongruent conditions are negatively correlated (and time synchronized). And a value for $\mathbf{C}^l_{R_j}(i, k)$ that is close to 0 indicates that the responses to congruent and incongruent stimuli conditions are uncorrelated between channel pairs (i, k).

For each language, these matrices are generated. A distance measure using Frobenius norm of $\mathbf{C}^1 - \mathbf{C}^2$ is computed for every pair of time regions. This generates the 5×5 distance matrix that is plotted in Figure 8 of the main article.

REFERENCES

Bae, G.-Y. and Luck, S. J. (2018). Dissociable decoding of spatial attention and working memory from eeg oscillations and sustained potentials. *Journal of Neuroscience* 38, 409–422

Coulson, S. and Wu, Y. C. (2005). Right hemisphere activation of joke-related information: An event-related brain potential study. *Journal of cognitive neuroscience* 17, 494–506

Kumar, M., Federmeier, K. D., and Beck, D. M. (2021). The n300: An index for predictive coding of complex visual objects and scenes. *Cerebral Cortex Communications* 2, tgab030

Kutas, M. and Federmeier, K. D. (2000). Electrophysiology reveals semantic memory use in language comprehension. *Trends in cognitive sciences* 4, 463–470

Meade, G., Lee, B., Midgley, K. J., Holcomb, P. J., and Emmorey, K. (2018). Phonological and semantic priming in american sign language: N300 and n400 effects. *Language, cognition and neuroscience* 33, 1092–1106

Picton, T. W., Bentin, S., Berg, P., Donchin, E., Hillyard, S., Johnson, R., et al. (2000). Guidelines for using human event-related potentials to study cognition: recording standards and publication criteria. *Psychophysiology* 37, 127–152

Schendan, H. E. and Kutas, M. (2002). Neurophysiological evidence for two processing times for visual object identification. *Neuropsychologia* 40, 931–945
